# Pyomelanin produced by *Streptomyces* sp. ZL-24 and its protective effects against SH-SY5Y cells injury induced by hydrogen peroxide

**DOI:** 10.1038/s41598-021-94598-3

**Published:** 2021-08-17

**Authors:** Yumei Li, Zhengmao Ye, Peng Lu, Lingchao Lu

**Affiliations:** 1 School of Bioscience and Biotechnology, University of Jinan, 336 West Road of Nan Xinzhuang, Jinan, 250022 China; 2 School of Materials Science and Engineering, University of Jinan, Jinan, China

**Keywords:** Biochemistry, Biological techniques, Biotechnology

## Abstract

A soluble melanin pigment produced by *Streptomyces* sp. ZL-24 was purified and named StrSM. The elemental analysis of StrSM showed it consists of carbon, hydrogen, and oxygen. The spectrum analysis, including ultraviolet–visible absorption spectrum, Fourier-transform infrared spectrum, and pyrolysis–gas chromatography–mass spectrometry, indicated that StrSM might be pyomelanin. High performance liquid chromatography and liquid chromatography–mass spectra analysis of intermediate metabolite showed the presence of homogentisic acid (HGA). Moreover, the enzyme 4-hydroxyphenylpyruvate dioxygenase, involved in HGA biosynthesis, showed high activity during melanin production. Subsequently, a tyrosinase gene (*mel*C2) and hydroxyphenylpyruvate dioxygenase gene double mutant demonstrated StrSM is pyomelanin. In vitro bioactivity assay showed that StrSM had excellent protective capability against SH-SY5Y cell oxidative injury. To our knowledge, the results firstly provide comprehensive data on Streptomyces pyomelanin identification and a promising candidate compound to treat oxidative injury of neurocytes.

## Introduction

Melanin is a type of negative charged heterogeneous pigment found in all biological kingdoms, including bacteria, fungi, plants, animals and humans^1–3^. It plays many self-protective roles, including anti-ultraviolet radiation, free radiation scavenging, chelating metal ions, and high temperature and drought tolerance^4–7^.

Melanin is classified into eumelanin, pheomelanin, and allomelanin, according to its chemical structure and biosynthesis pathways^8^. Eumelanin, mainly found in humans and microorganisms, is formed by oxidative polymerization of tyrosine and/or phenylalanine to L-DOPA, which is further converted into dopachrome then to melanin^9^. Pheomelanin results from cysteinylation of DOPA^10^. Allomelanins contain a heterogeneous group of polymers that include pyomelanin^11^.

Pyomelanin is produced by many strains during the catabolism of tyrosine or phenylalanine via the oxidation of HGA, including *Legionella pneumophila*, *Pseudomonas aeruginosa*, *Pseudoalteromonas lipolytica*, *Burkholderia cepacia*, *Shewanella algae*, *Vibrio cholera*, *Aeromonas media*, *Rubrivivax benzoatilyticus*^12–18^. The complete breakdown of tyrosine requires the activity of 4-hydroxyphenylpyruvic acid dioxygenase (4-HPPD) and HGA-oxidase^19^. In the absence of HGA-oxidase, or when HGA production exceeds the catalytic activity of HGA-oxidase, HGA accumulates and is excreted from the cell. These events result in auto-oxidation and self-polymerization of HGA, producing pyomelanin. In this case, deletion of the gene encoding HGA-oxidase results in hyperproduction of pyomelanin, while pyomelanin is absent when deletion of the gene encoding 4HPPD^13,14,18,20^.

Pyomelanin is a soluble bacterial pigment with a molecular weight ranging from 10 to 14 kDa^21^, which is smaller than other melanin pigments. Owing to the excellent metal binding, redox and electron shuttling properties, pyomelanin has been used in Fe(II) acquisition, metal and azo dye reduction, in situ metal contaminant immobilization and electron conduit^12^. It can also act as reporter genes and can be used as cosmetics, dyes, colorings, and sunscreens^22^. Therefore, the multifaceted nature of pyomelanin give it the potential for use in numerous applications.

In our previous study, soluble melanin produced by *Streptomyces* sp. ZL-24 was isolated, which exhibited strong antimicrobial, anti-biofilm, and antioxidant activities^23^.

Oxidative stress is the primary major causes of neurodegenerative diseases, including Alzheimer’s, Parkinson’s disease, and amyotrophic lateral sclerosis^24^. Reactive oxygen species (ROS) generated during normal cellular metabolism processes can be removed by intracellular antioxidant enzymes^25^. However, imbalances between ROS production and elimination that result from abnormalities of the antioxidant homeostasis system induce oxidative stress^26^. In this context, inhibiting ROS generation can protect normal neuronal cells from damage or death, thus prevent neurodegenerative diseases.

In this study, soluble melanin was isolated from *Streptomyces* sp. ZL-24. Subsequently, spectrum and biosynthesis pathway analyses identified it as pyomelanin, then its protective effects against oxidative injury of neurocytes were investigated in vitro.

## Materials and methods

### Melanin extraction and purification

The production and extraction of the soluble melanin pigments from *Streptomyces* sp. ZL-24 was performed using the method described in our previous study^23^. The protein was removed using pepsin and sevag reagent (chloroform/*n*-butyl alcohol, 4:1, v/v), and the nucleic acid fractions were digested by DNase I and RNase A. The extracted melanin pigments were then applied to Sephadex G-15 column (1 cm × 70 cm) with deionized water as eluent at a flow rate of 0.2 mL/min. Approximately 3 mL of the eluent flow was collected in each tube, and each fraction was measured at 520 nm^23^. The purified melanin pigment was then freeze-dried to obtain the pigment in powder form.

### Elemental analysis

The percentage contents of C, H, O, N (nitrogen), and S (sulfur) in melanin were measured using FlashSmart Elemental Analyzers (Thermo Fisher Scientific, USA).

### Ultraviolet–visible absorption spectrum

Melanin powder was dissolved in deionized water to a final concentration of 1 mg/mL. The UV–Vis spectrum was then recorded from 190 to 700 nm using a Cary 60 UV–Vis Spectrophotometer (Agilent, USA).

### Fourier-transform infrared spectrum

The mixture of melanin powder (2 mg) and dry Potassium bromide (KBr, 200 mg) was pressed into tablets, and FT-IR spectra were recorded between 4000 and 400 cm^−1^ using Bruker Vertex 70 FT-IR spectrometer (Bruker, Germany).

### Pyrolysis–gas chromatography–mass spectrometry analysis

Melanin was cleaved via single-step lysis by Frontier EGA/PY3030D thermal cracking apparatus (Frontier, Japan). The resulting lysate was analyzed by GC–MS QP2020 (Shimadzu, Japan), which was qualitatively searched using the National Institute of Standards and Technology (NIST) standard library. The detailed methods were as follows: the pyrolyzer was set at 650 °C, and the pyrolysis time was 15 s. The gas chromatography–mass spectra (GC–MS) analytical column was Rxi-5Sil MS 30 m × 0.25 mm × 0.25 μm. Meanwhile, the GC oven temperature was operated from 50 to 280 °C at a rate of 30 °C/min. MS transfer line temperature was 270°C, and electrospray ionization (EI) was employed. The ionization source temperature was 220 °C, the acquisition method was “scan”, and mass range was *m*/*z* 50–400 amu.

### Enzyme activity assay

The 4-HPPD activity was assayed in a final volume of 3 mL. The reaction mixtures consisted of 4-hydroxyphenylpyruvic acid (1 mM), ferrous sulfate (1 mM), and ascorbic acid (5 mM). The reaction was initiated by adding the cell-free supernatant to the appropriate reaction mixtures and incubating at 37 °C for 1 h. The reaction was stopped by adding 300 µL of 5N HCl. The reaction mixture was centrifuged at 12,000*g* for 10 min, and the resulting supernatant was used to detect the reaction product HGA.

### HPLC and LC–MS

Detection of HGA was achieved by HPLC equipped with an ultraviolet absorption detector. Samples were loaded directly onto an Agilent Eclipse Plus C18 reverse-phase column (5 μm particle size; 4.6 by 250 mm). The mobile phase was acetonitrile–deionized water (90:10 [vol/vol]), the flow rate was 1 mL/min. HGA was monitored at 290 nm, as previously described^26,27^.

Samples were run in an Agilent 6210 ESI-TOF LC/MS. Samples were run in negative-ion mode for 20 min. A formula confirmation method was used in the Agilent Mass Hunter software to detect HGA (formula C_8_H_8_O_4_). HGA (50 mM) was used as the standard, and retention times (Rt) of HPLC and MS were compared with those of the standard, respectively.

### Construction of gene deletion mutants

The homologous recombination method was employed to delete *hpp*D and *mel*C2 genes. The primers used to construct the mutants in supplementary Table S1.

To construct Δ*mel*C2, a 448 bp and a 504 bp homologous arms were amplified from strain ZL-24 genomic DNA with primers melC2up-F/melC2up-R and melC2down-F/melC2down-R, respectively. The resulting upstream and downstream PCR fragments (Fig. S1A) were assembled by overlapping PCR, then double-digested with HindIII and EcoRI. The digested fragments were then ligated on to pKC1139 digested with HindIII and EcoRI to obtain the plasmid pKC1139-∆*mel*C2, which was then conjugated into strain ZL-24 to obtain the strain ZL-24-Δ*mel*C2. Disruption of *mel*C2 was verified by PCR, confirming the deletion of a 545 bp fragment of the *mel*C2 gene (Fig. S1B).

To construct Δ*hpp*D, 1056 bp and a 951 bp homologous arms were amplified from the genomic DNA of strain ZL-24 with primers hppdup-F/hppdup-R and hppddown-F/hppddown-R (Fig. S2A), then double-digested with EcoRI and XbaI, and XbaI and HindIII, respectively. The digested fragments were then ligated with pKC1139 digested with EcoRI and HindIII to obtain the plasmid pKC1139-∆*hpp*D, which was then conjugated into strain ZL-24-Δ*mel*C2 to obtain the strain ZL-24-Δ*mel*C2/Δ*hpp*D. Disruption of *hpp*D was verified by PCR, confirming the deletion of a 1482 bp fragment of the *hpp*D gene (Fig. S2B).

### Complementation of *hpp*D deletion mutant

To complement the function of the deleted gene in strain ZL-24-Δ*mel*C2/Δ*hpp*D, the open reading frame of the *hpp*D gene was amplified through PCR from genomic DNA of strain ZL-24. The primers are shown in supplementary Table S1. PCR products were ligated to the plasmid pIB139 with an ermE promotor. The resulting complementary plasmid pIB139-*hpp*D was conjugated into the strain ZL-24-Δ*mel*C2/Δ*hpp*D to obtain an *hpp*D gene complementary strain (ZL-24-ChppD).

### Cell culture and treatment

Human neuroblastoma SH-SY5Y cells were cultured in high-glucose DMEM medium (Hyclone, USA) supplemented with 10% heat-inactivated fetal bovine serum (GIBCO, USA), 1% (*v/v*) penicillin (100 µg/mL) and 100 U/mL streptomycin at 37 °C with 95% humidified air and 5% CO_2_. Cells (1 × 10^4^ cells/mL) were seeded in 96-well plates. Upon reaching approximately 80% confluence, 1 × 10^6^ cell/mL cells were pretreated with different concentrations of StrSM (1, 5, 10, 30, 50, 100, 300 μg/mL) or H_2_O_2_ (50, 100, 200, 300, 400, 500, 600 μM) for 24 h at 37 °C. Subsequently, the cells were pre‑treated with various concentrations (1, 5, 10, 30, 50 μg/mL) of StrSM or *N*-acetylcystein (NAC) for 5 h and co-treated with 100 µM H_2_O_2_ for another 8 h at 37 °C. Based on Chen et al.’s study, 20 µM NAC was used as a positive control for antioxidant capacity that suppresses cell death^28^. SH-SY5Y cells were observed under the microscope (Olympus CKX53) with a magnification of 100 times.

### Cell viability assay

Cell viability was measured as follows: treated cells were plated in 96-well plates, precipitated for 1 h at 4 °C with 100 μL 10% trichloroacetic acid (Shenggong Biotech, China), and then stained with 50 μL sulforhodamine B (SRB; Sigma-Aldrich, USA). The optical density was recorded at 540 nm after reconstitution of the bound dye in 150 μL of 10 mM Tris base (pH 10.5) using a Spec-traMAX 190 microplate spectrophotometer (GMI Co., USA). Cell viability (%) = (OD of treated group/OD of the blank group) × 100^29^.

### Preparation of cell lysates

SH-SY5Y cells were adjusted to 1 × 10^6^ cell/mL density after trypsinization, washed twice using phosphate buffer, and centrifuged at 1000*g* for 10 min. The supernatant was then discarded. The cells were resuspended in phosphate buffer by adding a mixture of protease and phosphatase inhibitors (Roche Diagnostics, Basel, Switzerland), then disrupted by ultrasonic wave. The resulting cell lysates were collected via centrifugation at 12,000*g* for 5 min and used to the following measurement.

### Lactate dehydrogenase (LDH) activity assay

The LDH activity of the cell supernatant was assayed using a kit (Solarbio Life Sciences, China) according to the manufacturer’s instruction, which was based on the reaction that LDH can catalyze lactate to form pyruvate. The formed pyruvate can react with 2,4-dinitrophenylhydrazine to form pyruvate-dinitrophenylhydrazone, which can present maroon in alkaline solution, and it has an absorption maximum at 440 nm.

### Determination of malondialdehyde content

The content of cellular MDA was determined according to the thiobarbituric acid (TBA) test as previously described^30,31^. It is based on the reactivity of MDA with TBA to produce a red adduct which can determine the absorbance at 532 nm with a microplate reader.

### Determination of superoxide dismutase activity (SOD), and glutathione peroxidase activity (GSH-PX)

The SOD and GSH-PX activities were measured with a microplate reader (Thermo Fisher Co., USA) according to the instructions on the assay kit (Solarbio Life Sciences, China). The protein content was determined by the BCA Bradford protein assay (Solarbio Life Sciences, China).

### Measurement of ROS

The intracellular reactive oxygen species (ROS) level was measured by a nonfluorescent probe, 2′,7′-dichlorofluorescein diacetate (DCFH-DA), as previously described with some modification^32^. After treatment, SH-SY5Y cells (2 × 10^5^ cell/mL) were collected by centrifugation at 1000 g for 5 min, washed twice with phosphate buffer, incubated with DCFH-DA (10 μM) at 37 °C for 30 min in the dark. After incubation, the cells were washed three times with a medium. The fluorescence of DCFH was measured by flow cytometry (Cytoflex LX, Beckman Coulter, USA) with 488 nm/525 nm filters. The average fluorescence intensity analyzed using Flowjo software as an indicator of ROS.

### Statistical analysis

All experiments were performed in triplicate. The data are represented as mean ± standard deviation (SD). Significant differences were calculated in SPSS software (version 19.0) by one-way analysis of variance (ANOVA) and Dunnett post hoc test. *P* values less than 0.05 were considered statistically significant.

## Results and discussion

### Purification and elemental analysis

The soluble melanin produced by *Streptomyces* sp. ZL-24 was extracted with deionized water and purified via acid nuclease digestion, organic solvent treatment, and column chromatography. The purified melanin, named StrSM, was dried with a lyophilizer and used for elemental analysis.

Elemental analysis is an important method for the initial identification of melanin type, as it provides the main elements present, generally, C, H, N, S and O^7^. The elemental analysis of StrSM showed that the percentages of C, H, and O were 29.43%, 18.99%, and 49.57%, respectively. Neither N nor S was detected. Thus, the result excluded eumelanin because it contains N and pheomelanin, which has N and S^33^.

### UV–Vis spectrum

**Figure 1:**
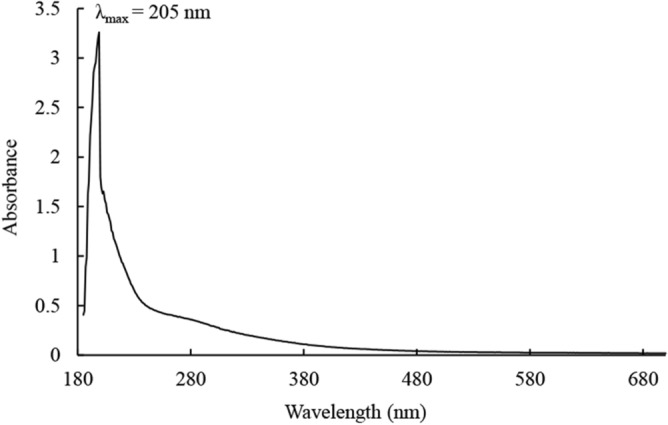
UV–Vis spectrum of StrSM.

As shown in Fig. 1, StrSM showed the maximum ultraviolet absorbance at 205 nm, and its optical density gradually decreased with wavelength increase. This result suggests that StrSM probably contains a conjugated double bond system or an aromatic ring structure. Meanwhile, no absorption peak was observed at 260 and 280 nm, indicating that StrSM did not contain nucleic acids or proteins^34^.

### FT-IR spectrum

**Figure 2:**
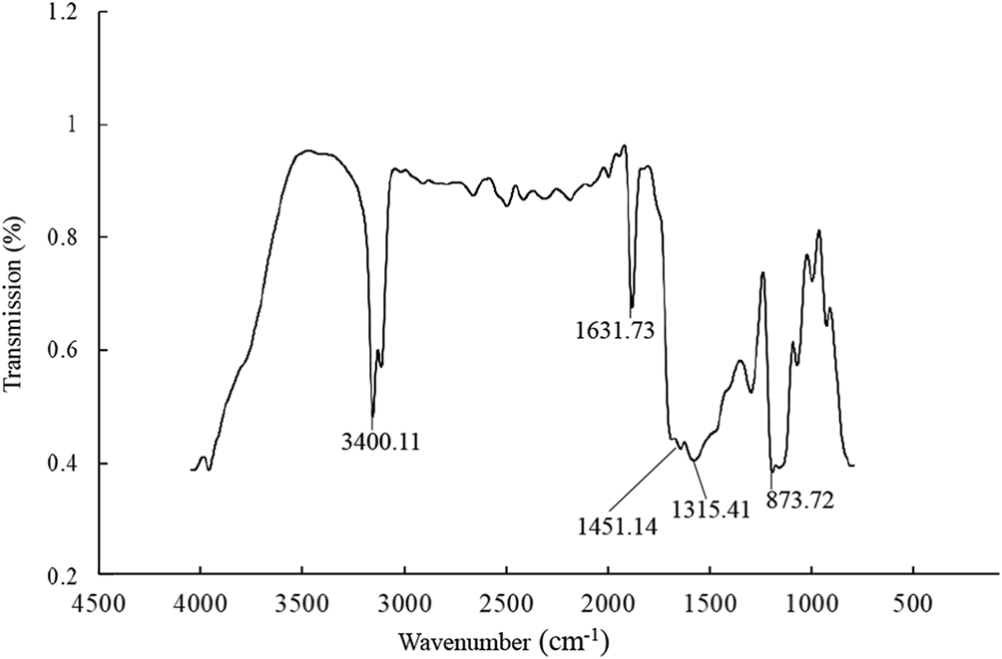
FTIR of spectrum of StrSM.

FT-IR provides information on the major functional groups in the compound. As shown in Fig. 2, the absorption peak at 3400.11 cm^−1^ could be assigned to the stretching vibration of an OH group^35^. Peaks observed at 1631.73 cm^−1^ and between 1451.14 and 1315.41 cm^−1^ were due to vibrations of aromatic ring C=C conjugated with C=O and/or COO− groups^36^ and OH groups of phenolics^37^. The peak around 873.72 cm^−1^ was due to the aromatic C–H group^38^. The FI-IR spectrum of StrSM observed in this study is similar to that of pyomelanin from *Penicillium chrysogenum*^39^.

### Py–GC/MS analysis

Py–GC/MS could provide new information about melanin structure. Py–GC/MS analysis of StrSM demonstrated that it was a complex heterogeneous polymer. The pyrolysis products of StrSM are shown in supplementary Table S2, which includes benzaldehyde, methoxybenzene and phenolic compounds identified using information in the NIST library (Lindstrom & Mallard). Based on the chemical composition, the major StrSM pyrolysis products could be divided into the following groups: Benzene (1–9), phenol (10–23), and their derivatives. The most abundant products were benzene and its derivatives, followed by phenol and its derivatives. Benzene, phenol, and their derivatives are the most prominent characteristic thermal degradation products of pyomelanin^33^. This result was consistent with that StrSM consisted of elements C, H, and O. Therefore, StrSM may be pyomelanin.

**Figure 3:**
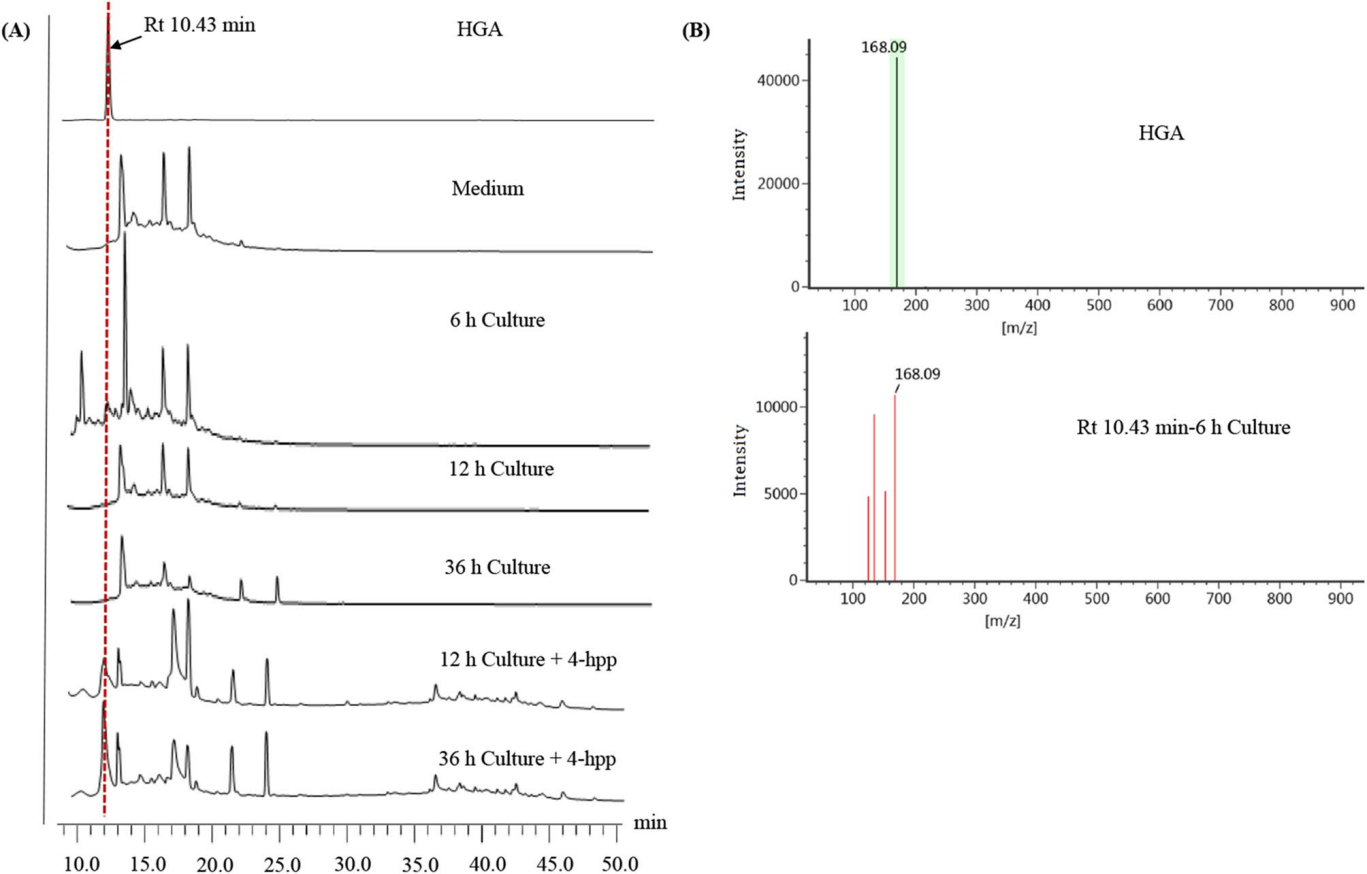
HPLC and MS analysis of HGA. (**A**) HPLC analysis. HGA is used as a standard; Medium is used as a negative control. 6, 12, and 36 h culture represent culture supernatant of strain ZL-24 at 6, 12, and 36 h. 12 h Culture + 4-hpp and 36 h Culture + 4-hpp represent the reaction mixtures of 12 h or 36 culture supernatant and 4-hpp. (**B**) MS analysis. HGA is used as a standard; Rt 10.43 min-6 h Culture represents the peak with Rt 10.43 min in HPLC profile of 6 h culture supernatant.

The monomeric precursor for pyomelanin synthesis was HGA, which was synthesized by 4-HPPD in vivo^20,40^. To determine the presence of HGA in metabolic intermediates, the cell-free supernatants from different cultivation time (6, 12, and 36 h), and standard HGA were subjected to HPLC analysis. The results showed that 6 h culture supernatant appeared the identical peak with HGA at retention time (Rt) of Rt 10.43 min, but no similar peaks in 12 h and 36 h culture supernatants, which may be caused by the fast transformation of HGA in the catabolism of the strain (Fig. 3A). Furthermore, it was desirable to study the activity of HPPD enzyme which catalyzes the conversion of 4-hpp to HGA. Thus, the reaction mixtures from the cell-free culture supernatants (12 h and 36 h) and 4-hydroxyphenylpyruvate (4-hpp) were further analyzed by HPLC, and HGA peaks were observed at Rt 10.43 min, which confirmed that the strain cultures had high HPPD enzyme activity (Fig. 3A). MS analysis of the peak with Rt 10.43 min in HPLC profile of 6 h culture supernatant showed a molecular ion mass of 168 [M +], which had mass fragmentation identical to that of HGA (Fig. 3B). These results demonstrated that strain ZL-24 can produce pyomelanin.

### Confirmation of pyomelanin biosynthesis

Based on our previous study, tyrosinase contributed to melanin synthesis in strain ZL-24^23^. Tyrosinase catalyzes tyrosine hydroxylation to L-DOPA, and its subsequent oxidation to *o*-dopaquinone. The *o*-dopaquinone then polymerizes to form melanin through a series of nonenzymatically and oxygen involved oxidoreduction reactions^41^. In this case, tyrosinase, encoded by *mel*C2 in the *Streptomyces* species^42^, was inactivated through deleting *mel*C2. The *mel*C2 deletion mutant (ZL-24-Δ*mel*C2) was inoculated in soybean peptone, LB, and tyrosine media to investigate the melanin biosynthesis pathway. Undifferentiated melanin production was observed between the wild-type strain and strain ZL-24-Δ*mel*C2 in the different media (Fig. S3), demonstrating that there were other biosynthesis pathways besides L-DOPA pathway.

**Figure 4:**
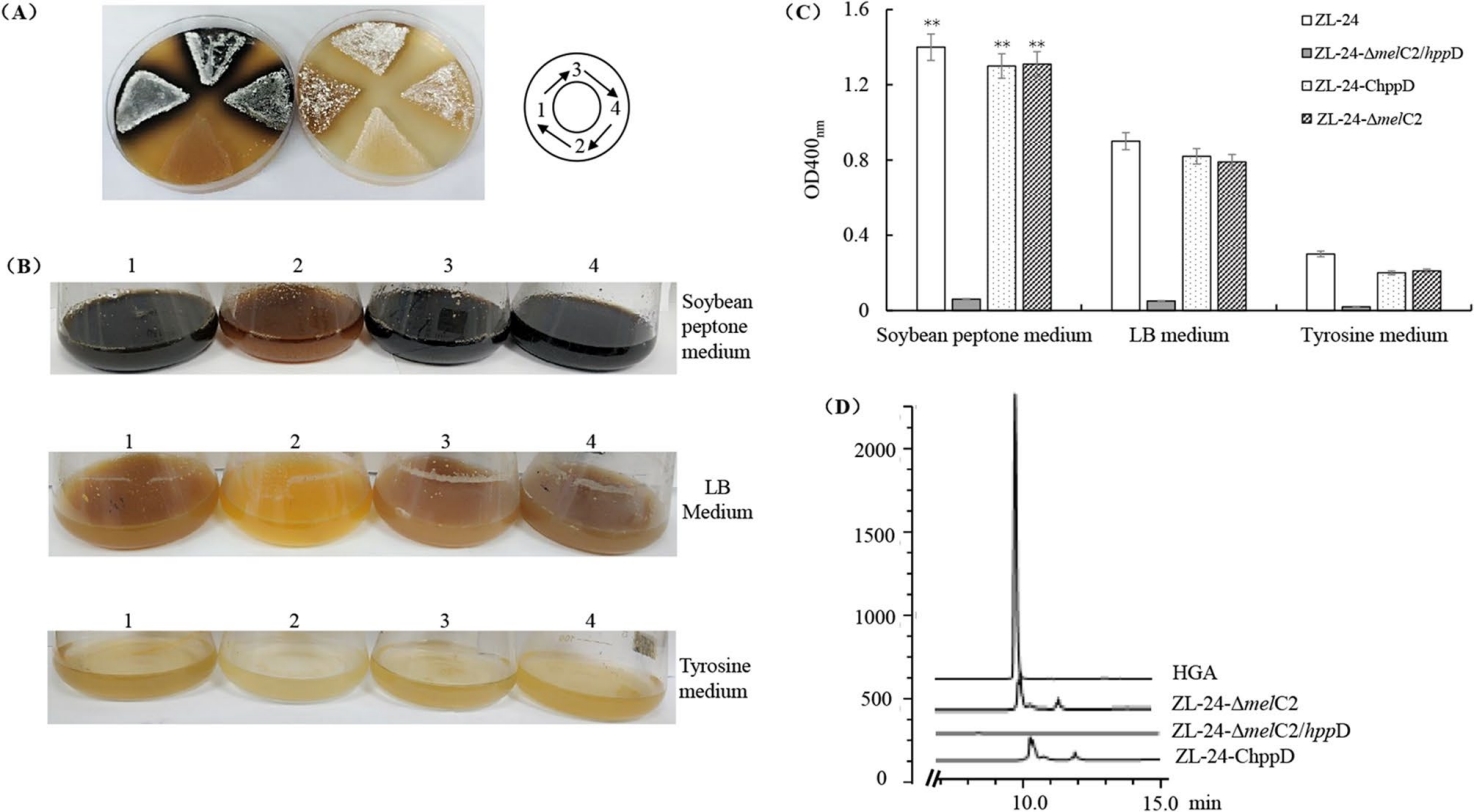
Characteristics of melanogenesis in wild-type ZL-24 (1), double deletion mutant ZL-24-Δ*mel*C2/*hpp*D (2), complementary strain ZL-24-ChppD (3), and ZL-24-Δ*mel*C2 (4). (**A**) Photographs of cultures at 60 h in culture on soybean peptone plate (left) and LB plate (right). (**B**) Photographs of cultures at 60 h in culture in soybean peptone medium (top), LB medium (middle), and tyrosine medium (bottom). (**C**) The melanin yield, as determined by OD400_nm_, after 60 h in culture in soybean peptone, LB and tyrosine media. Data are presented as mean ± standard deviation of three independent experiments. **p ≈ 0.004 < 0.01 is determined using one-way ANOVA and Dunnett post hoc test. (D) HPLC profiles of the cell free culture supernatant from ZL-24, ZL-24-Δ*mel*C2/*hpp*D, ZL-24-Δ*mel*C2, and ZL-24-ChppD at 6 h in culture in soybean peptone medium, HGA is used as a standard.

In bacteria, the HGA pathway also contributed to pyomelanin production, 4-HPPD converted 4-hpp to HGA. Therefore, deletion of the *hpp*D gene in strain ZL-24 could provide crucial information on the pyomelanin biosynthesis. This study further constructed a *mel*C2 and *hpp*D double deletion mutant (ZL-24-Δ*mel*C2/*hpp*D). The wild-type strain ZL-24 could produce melanin when grown in different media and cultivation times; ZL-24-Δ*mel*C2/*hpp*D completely lost pigmentation, suggesting that *hpp*D was involved in melanin synthesis of strain ZL-24 (Fig. 4A–C). As expected, introducing the plasmid pIB 139 carrying *hpp*D into the strain ZL-24-Δ*mel*C2/*hpp*D restored pigmentation of ZL-24-Δ*mel*C2/*hpp*D (Fig. 4A–C). As *hpp*D is predicted to convert 4-hpp to HGA, the culture supernatant of mutants with a deletion on hppD might not contain HGA. To test this, we employed HPLC to detect HGA in the 6 h culture supernatant of wild-type ZL-24, the deletion mutant ZL-24-Δ*mel*C2/*hpp*D and the complementary strain ZL-24-ChppD. The results showed that a peak matching the HGA standard was present in the culture supernatant of the wild-type strain, ZL-24-Δ*mel*C2 and ZL-24-Δ*mel*C2/hppD, but not in ZL24-Δ*mel*C2/*hpp*D (Fig. 4D). Therefore, these results provided strong support for the identity of StrSM as pyomelanin.

### Protective effects of StrSM on H_2_O_2_-induced cell injury in SH-SY5Y cells

**Figure 5:**
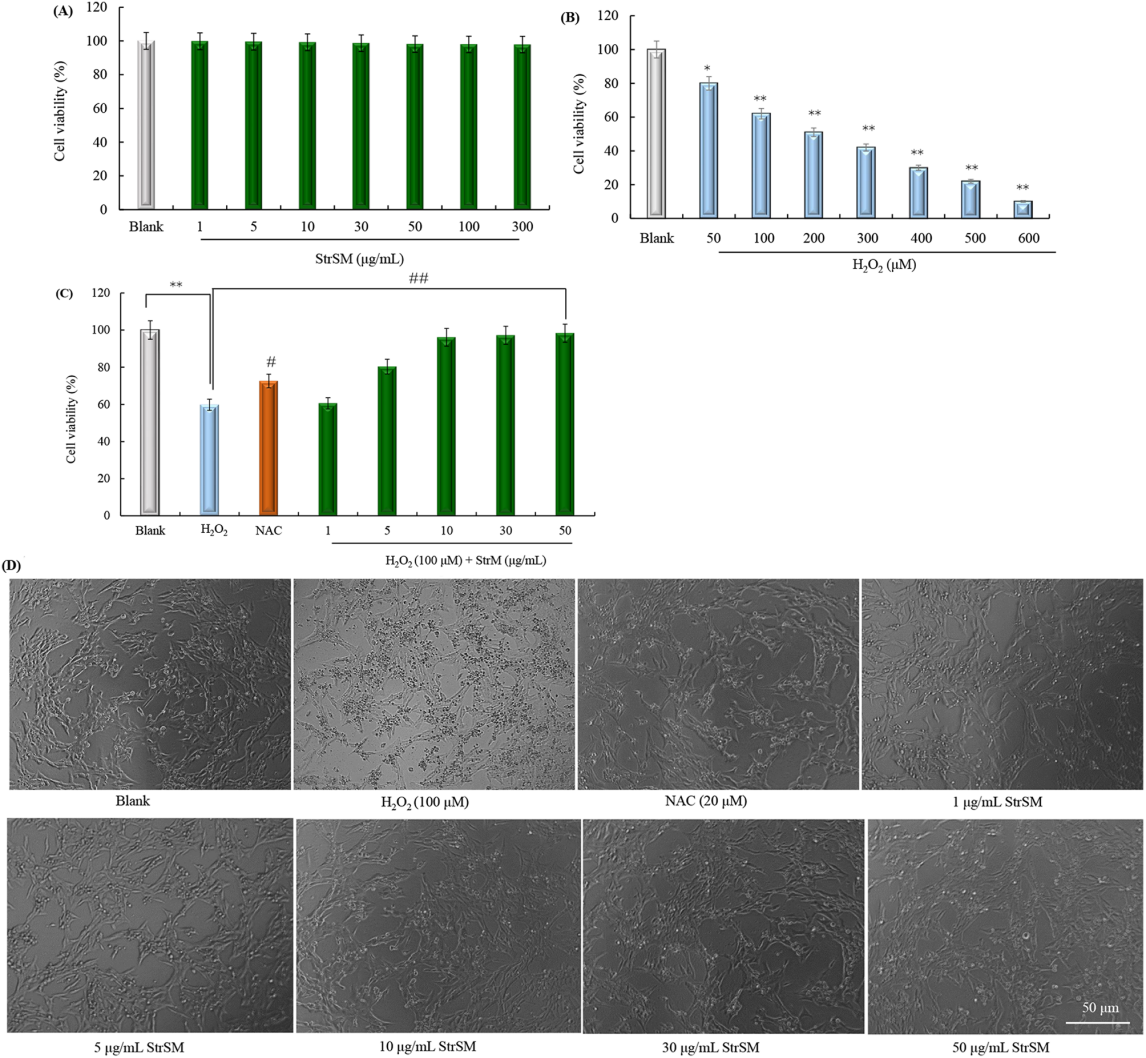
StrSM protects SH-SY5Y cells against H_2_O_2_-induced oxidative injury. (**A**) Cells are treated with various concentrations of StrSM. (**B**) Cells are treated with various concentrations of H_2_O_2_ for 24 h. (**C**) Cells are pre-treated with various concentrations of StrSM for 5 h followed by co-treatment with 100 µM H_2_O_2_ for 24 h and their viability. (**D**) The protective effect of StrSM on H_2_O_2_-induced morphological changes in SH-SY5Y cells. Cells are observed under 100 × magnification. Scale bar = 50 μm. Data are presented as mean ± standard deviation of three independent experiments. *p ≈ 0.02 < 0.05, ** *p ≈* 0.005 < 0.01, ^#^*p ≈* 0.03 < 0.05, and ^##^*p ≈* 0.002 < 0.01 are determined using one-way ANOVA and Dunnett post hoc test. NAC, *N*-acetylcystein; StrSM, melanin from *Streptomyces* sp. ZL-24.

SH-SY5Y cells are the most widely used human neuroblastoma cell line. This cell line exhibits some of the molecular and cellular processes of Parkinson’s disease (PD) and is thus used to study the therapeutic implications of PD in vitro models^43^. Exogenous H_2_O_2_ treatment has been used to induce oxidative stress that is related to neurodegenerative diseases^43^. In this study, SH-SY5Y cells were used to determine the effects of StrSM treatment on cell viability. The results showed treatment with these concentrations of StrSM alone did not affect cell viability and caused no cell toxicity (Fig. 5A). Subsequently, the effect of H_2_O_2_ (50–600 µM) on SH-SY5Y cells was evaluated, which indicated that H_2_O_2_ reduced cell viability in a concentration-dependent manner. Incubating 100 µM H_2_O_2_ with SH‑SY5Y cells for 24 h was confirmed to be an appropriate condition for inducing oxidative stress injury in the SH-SY5Y cellular model in vitro, resulting in approximately 60% cell viability (Fig. 5B). In parallel, the cell viability increased to 61.6%, 80.3%, 96.2%, 97.3%, 98.1% or 72.4% when cells were pre-treated with StrSM (1, 5, 10, 30, and 50 µg/mL) or NAC, respectively (Fig. 5C). At the meantime, the protective effect of StrSM was also evidenced by morphologic observations of SH-SY5Y cells. Cells exposed to H_2_O_2_ developed distorted irregular shapes or underwent cell death. Meanwhile, cell co-treatment with H_2_O_2_ and StrSM or NAC generated minimal morphological changes (Fig. 5D). These data demonstrate that StrSM pretreatment prevented H_2_O_2_-induced cell injury and morphological changes, whereas 100 μM H_2_O_2_ alone decreased cell viability. Therefore, StrSM had a neuroprotective effect on H_2_O_2_-treated SH-SY5Y cells.

### StrSM protects H_2_O_2_-induced cell injury in SH-SY5Y cells

**Figure 6:**
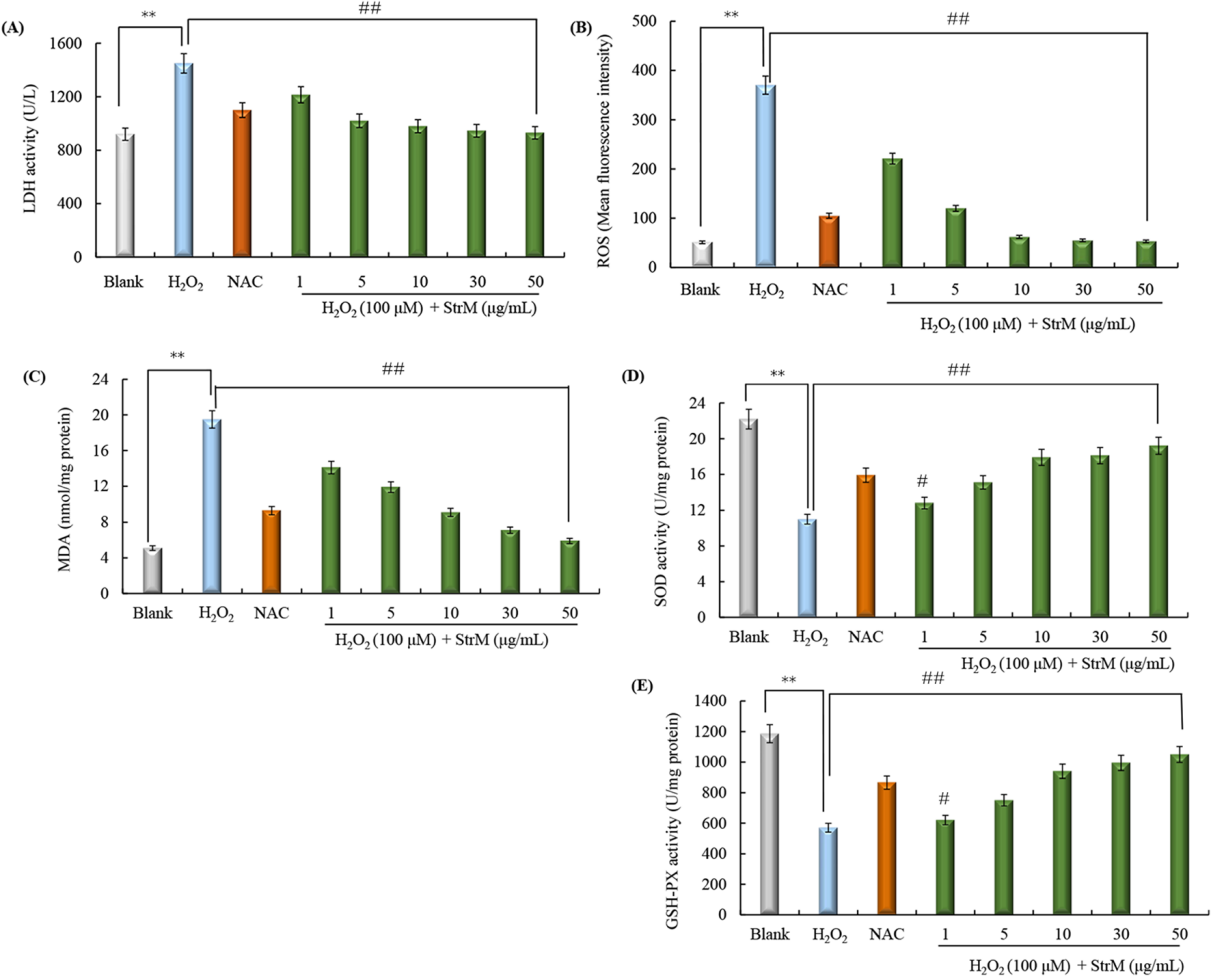
Effects of StrSM on LDH activity (**A**), intracellular ROS accumulation, (**C**) MDA content, (**D**) SOD, and (**E**) GSH-PX activity in SH-SY5Y cells. SH-SY5Y cells are pre-treated with different concentrations of StrSM (1, 5, 10, 30, and 50 μg/mL) for 5 h followed by co-treatment of 100 μM H_2_O_2_ or 20 μM NAC for 24 h. The model group is only treated with 100 μM H_2_O_2_ for 24 h. Data are presented as mean ± standard deviation of three independent experiments. **p ≈ 0.006 < 0.01, ^#^*p ≈* 0.04 < 0.05, and ^##^*p ≈* 0.005 < 0.01 are determined using one-way ANOVA and Dunnett post hoc test. *NAC N*-acetylcystein, *StrSM* melanin from *Streptomyces* sp. ZL-24.

The LDH release and ROS generation are able to reflect cell damage caused by oxidative stress. Thus, this study measured LDH activity and ROS production to confirm the protective effects of StrSM against oxidative stress-induced cell injury^44^. As shown in Fig. 6A, 100 µM H_2_O_2_ significantly increased LDH activity. However, pre-treatment with StrSM or NAC, reduced the amount of LDH release. Similarly, H_2_O_2_ treatment caused a marked increase of intracellular ROS generation; however, 1–50 μg/mL StrSM pretreatment significantly reduced ROS production (Fig. 6B). Therefore, StrSM could reduce cellular LDH activity and ROS production that caused by H_2_O_2_ exposure.

To clarify how StrSM protects against SH-SY5Y cell oxidative injury, the MDA content, SOD, and GSH-PX activities were measured. The results indicated that H_2_O_2_ caused a remarkable increase in MDA content and decrease in SOD and GSH-PX activities. However, pre-treatment with StrSM (1, 5, 10, 30, and 50 µg/mL) significantly reduced the increase in MDA content induced by H_2_O_2_ (Fig. 6C), while it significantly reversed the decrease in SOD and GSH-PX activities induced by H_2_O_2_ (Fig. 6D,E). These data mean that StrSM exerted protective effects against SH-SY5Y cell oxidative injury induced by H_2_O_2_ exposure by decreasing the MDA content and increasing SOD and GSH-PX activities.

## Conclusions

In this study, a soluble melanin pigment produced by *Streptomyces* sp. ZL-24 (StrSM) was identified through its elemental composition, spectrum characteristics, and biosynthesis pathway. Then, cell viability and the protective effect of StrSM on human neuroblastoma SH-SY5Y cell oxidative injury were investigated. The results and conclusions are as follows: (1) the element and spectrum analysis showed that StrSM might be pyomelanin. (2) The deletion mutants of *mel*C2 and *hpp*D genes indicated that StrSM was synthesized through the HGA-pathway, which demonstrated StrSM is pyomelanin. (3) StrSM exhibited excellent neuroprotective effects in SH-Y5Y cells, suggesting that StrSM may be a new candidate compound to treat neurodegenerative diseases.

## Supplementary Information

Supplementary Informations.
