# Supplementary material for: Pyomelanin produced by Streptomyces sp. ZL-24 and its protective effects against SH-SY5Y cells injury induced by hydrogen peroxide
Source: Sci Rep. 2021 Aug 17;11:16649. doi: 10.1038/s41598-021-94598-3 (PMC8371117; doi:10.1038/s41598-021-94598-3)
Supplement: Supplementary file 1 — Supplementary Informations. [file 41598_2021_94598_MOESM1_ESM.pdf]

Pyomelanin produced by *Streptomyces* sp. ZL-24 and its protective effects against SH-SY5Y cells injury induced by hydrogen peroxide

Yumei Li [1*], Zhengmao Ye[2*], Peng Lu[1], Lingchao Lu[2]

[1]School of Bioscience and Biotechnology, University of Jinan, Jinan, China

[2]School of Materials Science and Engineering, University of Jinan, Jinan, China

Address correspondence to Yumei Li, School of Bioscience and Biotechnology, University of Jinan, 336 West Road of Nan Xinzhuang, 250022, Jinan China.

E-mail: mls_liym@ujn.edu.cn, mse_yezm@ujn.edu.cn

Supplementary Materials

Table S1. Primers in this study.

Primers	Sequence 5'—3'	Amplified fragment
melC2up-F	ATCTAAGCTTTCTGGCCGCCCACCCACACG	Upstream of *mel*C2
melC2up-R	AGCCTGACCGCCGCCGAGAAG	
melC2d-F	CTTCTCGGCGGCGGTCAGGCTAAAGTCCCCCC AAGTGCGGCAG	Downstream of *mel*C2
melC2d-R	ATCGGAATTCCACGCTGGTGAACGTCACCGTG	
melC2test-F	GTCCCCCCAAGTGCGGCAG	fragment internal to *mel*C2 gene
hppdup-F	CCG GAATTC ATGCACTGGAAGACCGCGCTGA CGT	Upstream of *hpp*D
hppdup-R	CTAG TCTAGA GTTCGAAGGCTCTCCCCGCAT	
hppddown-F	CTAG TCTAGAGCCGTACGTATCCCTCACACGAC	downstream of *hpp*D
hppddown-R	ATCC AAGCTT ACCAGAGCGCGCGTCACCAAG	
hppdtest-F	GGTGGTCAGCCTGTACAGAT	fragment internal to *hpp*D gene
M13-R	CAGGAAACAGCTATGAC	
hppd-F	AATTCATATG ATGACTGAGACTCTGCACAC	*hpp*D
hppd-R	AATTCCGCGG TCAGAGGTTGCCGCGCTTCTC CTGCTC	

Table S2. Main pyrolysis products of StrSM

No.	Compound Structure	Compound Designation	No.	Compound Structure	Compound Designation
1	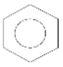	Benzene	13	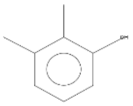	2,3-dimethyl Phenol
2	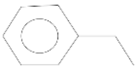	Ethylbenzene	14	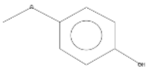	4-methoxy-Phenol
3	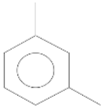	m-Xylene	15	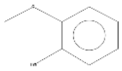	2-methoxy-Phenol
4	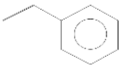	Styrene	16	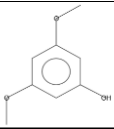	3,5-dimethoxy-Phenol
5	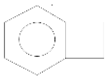	Benzocyclobutene	17	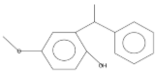	4-Methoxy-2-(1-phenylethyl) phenol
6	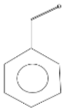	Benzaldehyde	18	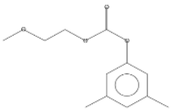	Carbonic acid, 2-methoxyethyl 3,5-dimethylphenyl
7	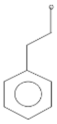	Benzeneacetaldehyde	19	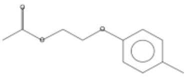	Ethanol, 2-(4-methylphenoxy)-, acetate
8	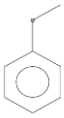	MethoxyBenzene	20	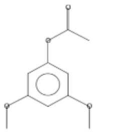	Phenol, 3,5-dimethoxy-, acetate
9	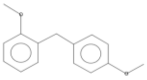	1-methoxy-2-[(4-methoxyphenyl)methyl]-Benzene	21	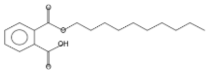	2-(Decyloxy carbonyl)-benzoic acid
10	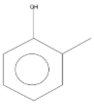	2-methyl-Phenol	22	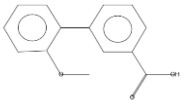	3-(2-Methoxyphenyl) benzoic acid
11	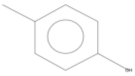	4-methyl-Phenol	23	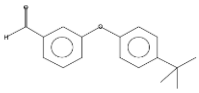	Benzaldehyde, 3-[4-(1,1-dimethylethyl) phenoxy]
12	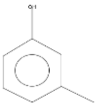	m-Cresol			

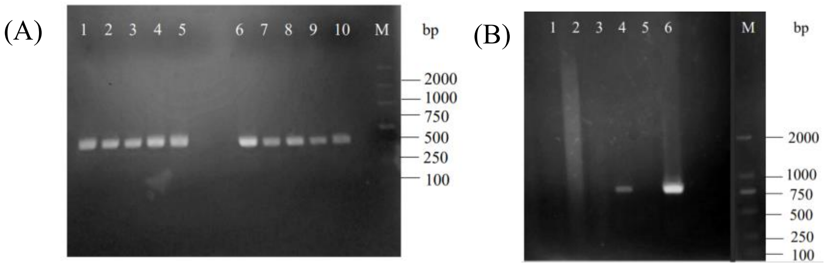

Fig. S1 (A) 1-5 and 6-10 represent upstream and downstream PCR fragments of *mel*C2 gene, respectively; M represents DNA ladder (100, 250, 500, 750, 1000, 2000 bp). (B) 1-5 represents PCR screening of *mel*C2 gene deletion; 6 represents PCR fragments of *mel*C2 gene.

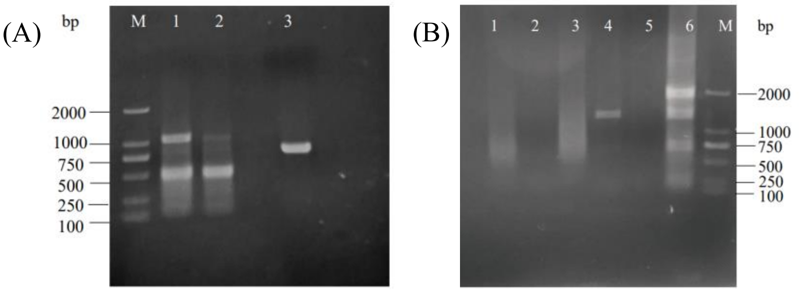

Fig. S2 (A) 1-5 and 6-10 represent upstream and downstream PCR fragments of *hpp*D gene, respectively; M represents DNA ladder (100, 250, 500, 750, 1000, 2000 bp). (B) 1-5 represents PCR screening of *hpp*D gene deletion; 6 represents PCR fragments of *hpp*D gene.

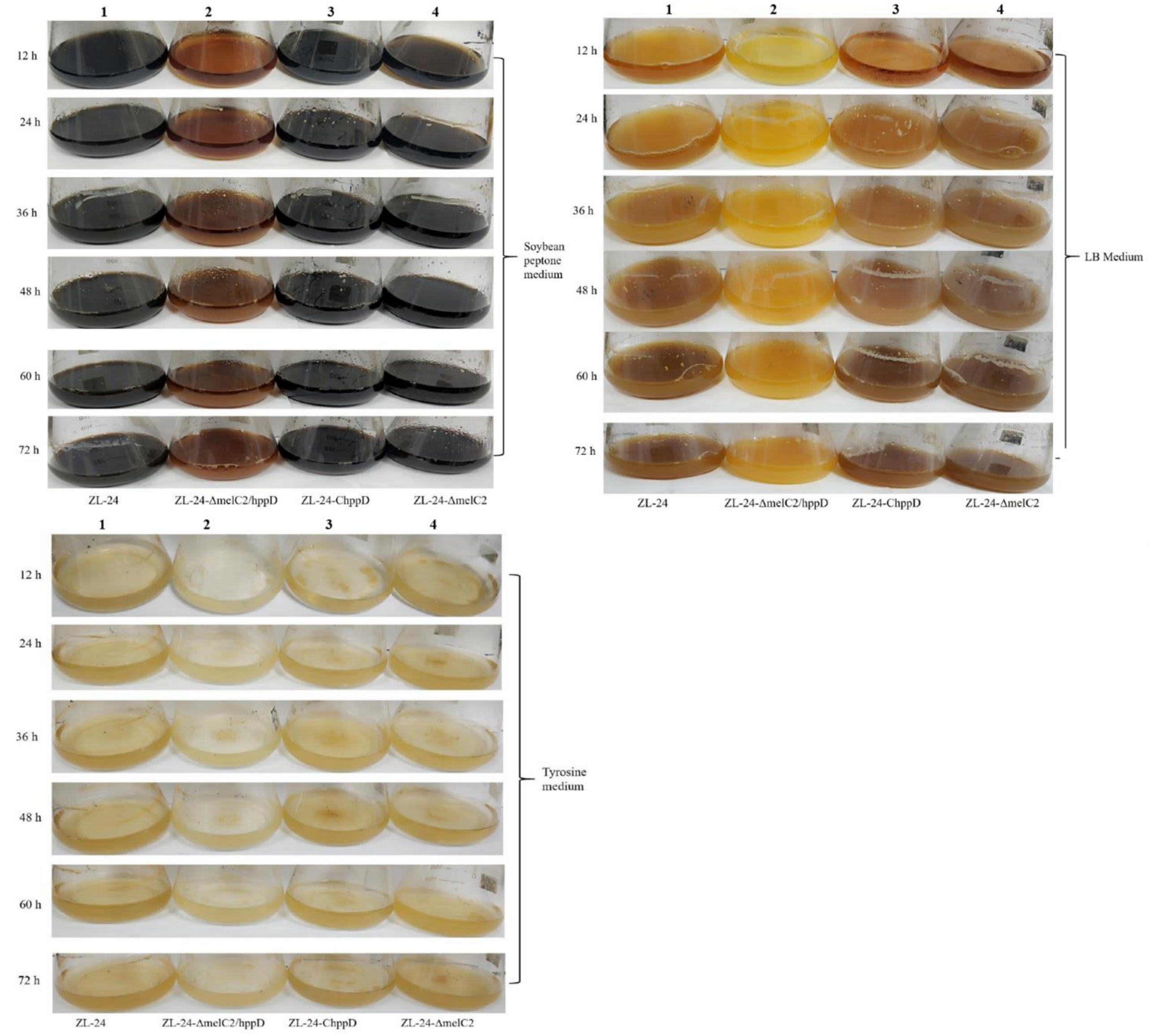

Fig. S3 Characteristics of melanogenesis in wild-type ZL-24 (1), double deletion mutant ZL-24-Δ*mel*C2/*hpp*D (2), complementary strain ZL-24-ChppD (3), and ZL-24-Δ*mel*C2 (4) in soybean peptone, LB, and tyrosine media at different cultivation time (12, 24, 36, 48, 60, 72 h).
